# Disseminated Hypervirulent Klebsiella pneumoniae Infection Following Travel: A Case of Cavitating Pneumonia, Hepatic and Renal Abscesses, and Thrombosis

**DOI:** 10.7759/cureus.82059

**Published:** 2025-04-11

**Authors:** Konstantinos Baronos, Simon Scott, Christopher Hebbes

**Affiliations:** 1 Medicine, University Hospitals of Leicester NHS Trust, Leicester, GBR; 2 Anesthesia and Intensive Care Medicine, University Hospitals of Leicester NHS Trust, Leicester, GBR

**Keywords:** cavitating pneumonia, community-acquired infections, diabetic ketoacidosis (dka), hypervirulent klebsiella pneumoniae (hvkp), klebsiella pneumoniae (kp), multidrug resistance, thromboembolic complications

## Abstract

Hypervirulent *Klebsiella pneumoniae* (hvKP) is a recently emerging pathogen that causes severe community-acquired infections in immunocompetent patients, in contrast to classical *K. pneumoniae,* which is found in nosocomial settings. We report the case of a healthy 55-year-old woman who, following recent travel to Singapore, presented with diabetic ketoacidosis (DKA) and septic shock. She presented with fever, cough, myalgias, and confusion, imaging demonstrating bilateral cavitating pneumonia, hepatic and perinephric abscesses, and renal vein and inferior vena cava thrombosis. Whole-genome sequencing identified hvKP (ST420, K2 capsular type, rmpA, rmpA2). The patient required admission to the intensive care unit (ICU) for mechanical ventilation, broad-spectrum antibiotics, and anticoagulation, and, despite progress on a stepwise incline, irreversible cavitating lung necrosis necessitated prolonged ICU dependence (>35 days). This case is notable for hvKP's virulence, its relation to travel to endemic regions, and the impact of diabetes on susceptibility, underscoring the need for early diagnosis, targeted therapy, and scrupulous source control.

## Introduction

Hypervirulent *Klebsiella pneumoniae* (hvKP) is a recently emerging pathogen responsible for severe community-acquired infections, including pneumonia, abscesses, thrombosis, central nervous system infection, and endophthalmitis. Unlike classical *Klebsiella pneumoniae* (cKP), which predominantly infects immunocompromised hosts, hvKP is more virulent due to genes such as rmpA and rmpA2, which enable capsule biosynthesis and immune evasion. First reported in East Asia, hvKP has since been identified from across the globe, raising concerns about its rapid dissemination and evolving antimicrobial resistance [1].

Virulence of the bacterium is driven by processes such as biofilm, a compact polysaccharide capsule, and siderophore-mediated iron acquisition, promoting immune evasion and hematogenous spread. These processes increase the potential for metastatic infection, particularly in diabetes mellitus, where hyperglycemia enhances capsule production and bacterial growth. Moreover, hvKP has been correlated with thromboembolic complications due to the fact that it is able to trigger disruption of vascular integrity, thereby contributing to the severity of the disease [1-4].

The current case report describes a disseminated hvKP infection in an otherwise healthy patient, highlighting the pathogen's aggressive nature and diagnostic challenges. Given the increasing prevalence of multidrug-resistant hvKP strains, early recognition, rapid intervention, and targeted antimicrobial therapy are critical in improving patient outcomes.

## Case presentation

A 55-year-old Indian female with no significant past medical history presented to the emergency department (ED) following recent travel to Singapore, with a 10-day history of fever, cough, myalgias, reduced oral intake, and a recently resolved urinary tract infection treated with trimethoprim. On initial evaluation, the patient had diabetic ketoacidosis (DKA) with hyperglycemia, Kussmaul breathing, and confusion. On examination, chest auscultation revealed bilateral crackles, and imaging showed bilateral patchy infiltrates. Blood tests revealed elevated white cell count, C-reactive protein, and procalcitonin, suggesting bacterial pneumonia. The patient was thus admitted to the intensive care unit (ICU) and intubated due to worsening type 2 respiratory failure (Table 1). 

**Table 1:** Summary of key blood test results obtained on hospital admission. HbA1C: hemoglobin A1C; pCO_2_: partial pressure of carbon dioxide; pO_2_: partial pressure of oxygen

Blood test	Value	Reference Range	Units
White Cell Count	35.6	4.0 - 11.0	x10^9/L
Neutrophil Count	30.97	1.5 - 7.5	x10^9/L
Procalcitonin	17.53	<0.05	ng/ml
C-reactive Protein	319	0 - 10	mg/L
Glucose Levels	33	5.6 - 6.9	mmol/l
HbA1C	15.9	4.0 - 5.9	%
pH	7.124	7.35 - 7.45	
pCO_2_	4.08	4.6 – 6.4	kPa
pO_2_	4.39	11.0 – 14.4	kPa
Bicarbonate	10.7	22 -29	mmol/l
Anion Gap	22.3	6 - 16	mmol/l

Extended cultures of respiratory samples grew *Klebsiella pneumoniae* positive, while negative for tuberculosis and *Aspergillus*, and negative blood cultures. Extended respiratory polymerase chain reaction (PCR) panels excluded viral pathogens (SARS-CoV-2, influenza, and respiratory syncytial virus (RSV)). The immunological workup came back negative for vasculitis (negative anti-neutrophil cytoplasmic antibodies (ANCA), antinuclear antibodies (ANA), proteinase 3 (PR3), myeloperoxidase (MPO)) and autoimmune triggers. Whole genome sequencing later confirmed hvKP: ST420 lineage, K2 capsular type, positive for virulence markers rmpA and rmpA2.

**Figure 1:**
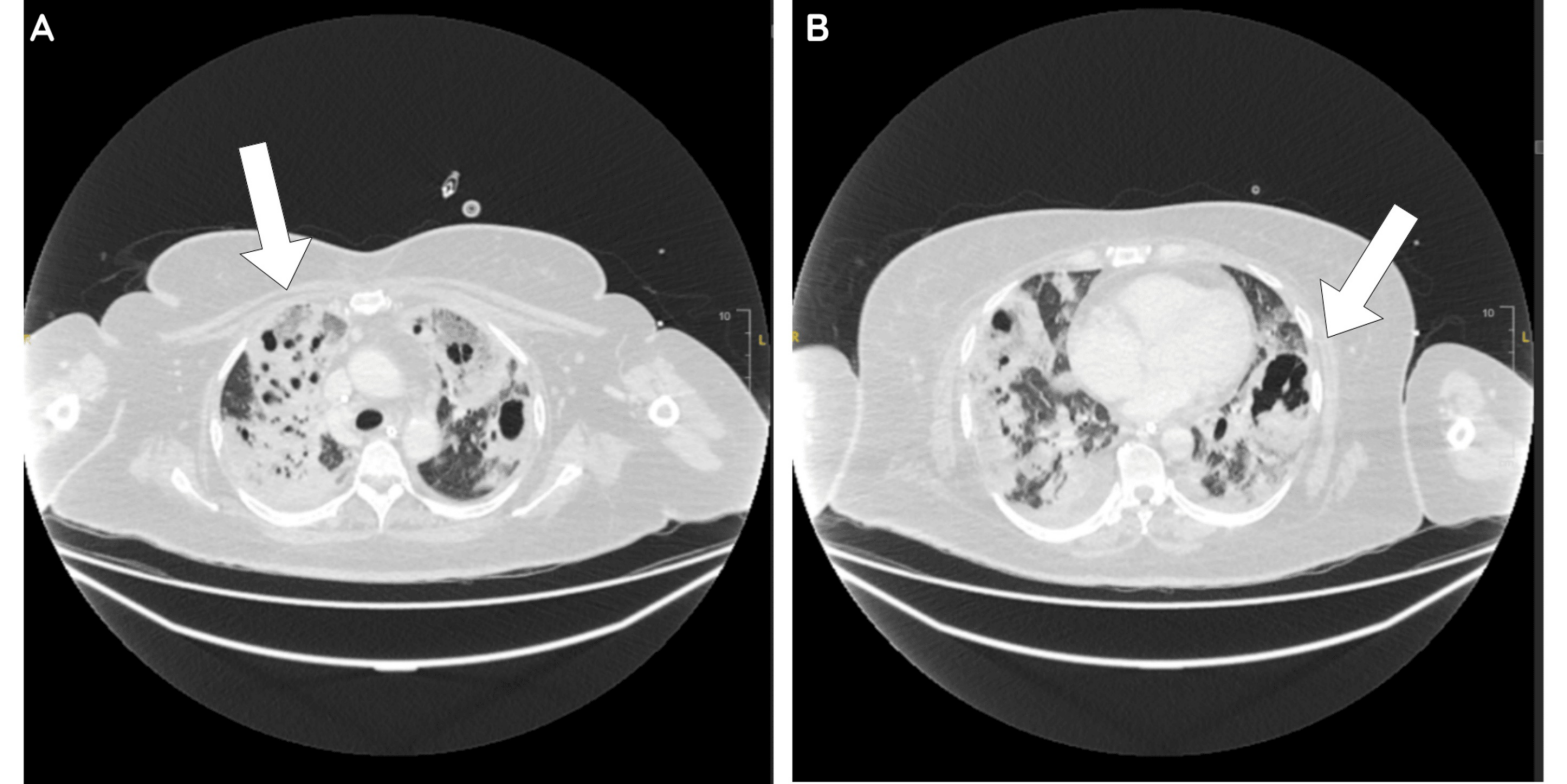
Computed tomography (CT) scan of the chest showing dense bilateral cavitating pulmonary consolidations, with arrows pointing to the cavities. Figure A shows multiple cavities in the right upper lobe, and Figure B depicts cavities in the left lower lobe.

**Figure 2:**
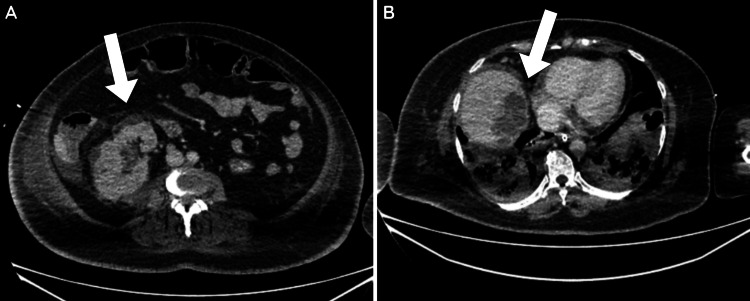
Computed tomography (CT) scan of the abdomen shows abscesses in the hepatic dome and right perinephric region. Figure A shows the right perinephric abscess, and Figure B shows the liver abscess, with arrows pointing to the respective abscesses.

Initial chest X-ray imaging showed bilateral diffuse and patchy ill-defined changes with a degree of nodularity. Subsequent computed tomography (CT) of the chest, abdomen, and pelvis revealed dense bilateral cavitating pulmonary consolidation representing the main septic focus (Figure 1). Secondary abscesses were detected in the hepatic dome and right perinephric region (Figure 2), and right renal vein and inferior vena cava (IVC) thrombosis. Ultrasound-guided drainage of the lung abscess was not possible due to anatomical challenges, including the location and the number of cavities present. A repeat imaging follow-up demonstrated progressive cavitating pneumonia, without any significant change in the extent of hepatic and right renal collections.

Treatment consisted of broad-spectrum antibiotics (meropenem, ciprofloxacin, later switched to piperacillin-tazobactam) and antifungals (voriconazole) for concomitant *Candida albicans* in tracheal aspirates, continuous veno-venous hemofiltration (CVVH) for acute kidney injury, vasopressors, and anticoagulation with heparin. The patient exhibited gradual, albeit modest, clinical improvement, characterized by reduced fraction of inspired oxygen (FiO₂) requirements and successful tolerance of continuous positive airway pressure (CPAP)/airway pressure release ventilation (APRV) trials. The patient remained ventilator-dependent in the ICU due to irreversible cavitating lung necrosis, and at the time of this report, the patient is in the ICU with a prolonged stay of 35 days. 

The case highlights a severe presentation of hvKP with metastatic abscess formation and thrombosis in a previously healthy traveler, complicated by delayed source control and prolonged critical illness. 

## Discussion

We report a case of hvKP infection in an immunocompetent patient who developed metastatic abscess formation in the liver and kidney and thromboembolic complications, underlining the aggressive nature of this pathogen and pointing out the challenge in managing the systemic manifestations. This discussion delves into the molecular mechanisms of virulence, the interplay of host factors, and the evolving epidemiology of hvKP, contextualizing the case within broader scientific and clinical paradigms [1].

Its pathogenesis, in this case, is attributed to the virulence factors of hvKP. Whole-genome sequencing confirmed the presence of the hypervirulent ST420 lineage with K2 capsular type and positive for virulence markers rmpA and rmpA2. The rmpA and rmpA2 biomarkers enhance capsule polysaccharide production, which protects the bacterium from phagocytosis, neutrophil extracellular traps (NETs), and complement-mediated activity [2-5], and the capsule type of K2 has been linked to metastatic spread in hvKP [6]. In vitro studies have shown that *Klebsiella pneumoniae* increases capsule biosynthesis when present in glucose-rich environments via reduction of cyclic adenosine monophosphate (cAMP) levels, thus protecting the bacteria from serum killing and enhancing the virulence of hvKp in diabetic patients [3]. Furthermore, there have been several factors identified in the literature that promote the growth and survival of hvKP, such as the production of siderophores sequestering iron from the host, enabling the bacterium to thrive in iron-limited host environments [7], colibactin (a genotoxic molecule) improving the survival in the bloodstream [8], and PEG344, part of the virulence plasmid and specific to hvKP, which, although the mechanism is unknown, is believed to be required for the full virulence and survival of Klebsiella [2,9].

The hvKP commonly colonizes mucosal surfaces such as the gastrointestinal and respiratory tracts, where it can persist as a reservoir for invasive disease. Biofilm formation, characterized by the secretion of an extracellular polymeric substance (EPS) that encapsulates bacterial communities, enhances adherence to mucosal surfaces even in the absence of indwelling medical devices. This EPS barrier interferes not only with host immune responses by restricting phagocytosis and protecting the bacteria from complement-mediated lysis but also with the significant reduction of the penetration of antimicrobials [10,11].

The spread of hvKP in this case likely occurred hematogenously, as evidenced by the development of distant abscesses in the liver and kidney and the presence of thrombosis. The right renal vein and IVC thrombosis observed in this patient underscores hvKP's unique ability to disrupt vascular integrity. Beyond the procoagulant effects of systemic inflammation, hvKP's capsule and lipopolysaccharide (LPS) directly activate endothelial cells, leading to the upregulation of tissue factors and promoting platelet aggregation [12]. In addition, hvKP may induce the formation of NETs, providing a matrix that traps platelets and fibrin, resulting in the formation of a thrombus [4,5,13,14]. These NETs, while an essential component of the innate immune response, can inadvertently exacerbate coagulation, contributing to septic thrombosis. 

Host predisposition factors played a role in the severity of this infection. The patient’s poorly controlled diabetes mellitus, evidenced by a hemoglobin A1C (HbA1c) of 15.9%, is a well-documented risk factor for hvKP infections. Poor glycemic control has been linked to an increase in metastatic spread of the disease [15], and a plausible hypothesis is the loss of vascular integrity, creating a permissive environment for bacterial spread [1]. Additionally, the patient’s Southeast Asian ethnicity may have played a role, as hvKP is endemic in this region. Although more evidence is needed to address whether a genetic variation or environmental exposure increases susceptibility, hvKP infections are more prevalent in Asians, Hispanics, and Pacific Islander individuals [1,16].

While this isolate remained susceptible to most β-lactams, the resistance to amoxicillin underscores the growing threat of antimicrobial resistance in hvKP. The hypervirulence and multidrug resistance of hvKP are believed to be driven by horizontal gene transfer between hvKP and cKp [17]. Carbapenem-resistant hvKP strains have already emerged in Asia, further narrowing the therapeutic window [18]. This convergence of hypervirulence and multidrug resistance not only complicates treatment strategies but also heightens the risk of rapid disease progression and poor patient outcomes. 

Despite appropriate antimicrobial therapy, the undrainable abscesses and progressive pneumonia of the patient point to some limitations in current treatments. Phage therapy has gained significant attention as an alternative to antibiotics, with studies identifying phages effective against certain types of capsules (particularly K1 or K5), but not all [19]. Although this is still in development, there have been a variety of scientific and regulatory concerns [20]. 

## Conclusions

Hypervirulent *Klebsiella pneumoniae* is a rapidly emerging pathogen with the potential for severe systemic infections, even in previously healthy individuals. The case illustrates the importance of early detection and targeted treatment because hvKP's enhanced virulence factors facilitate immune evasion, metastatic spread, and thromboembolic complications. Underlying disease processes, such as diabetes mellitus, predispose patients to a higher risk, illustrating the need for heightened clinical awareness. With the rising prevalence of multidrug-resistant strains, conventional antibiotic treatments may be insufficient, necessitating research into alternative therapies such as bacteriophage treatment. Improved diagnostic methods, early intervention, and stringent infection control measures will be instrumental in reducing the global burden of hvKP. Continued research and surveillance are critical to developing effective treatment strategies and reducing the burden of this increasingly prevalent pathogen.
